# Which Patients in the FLS Should Be Prioritised for a DXA Scan Within 12 Weeks?

**DOI:** 10.3390/jcm14165619

**Published:** 2025-08-08

**Authors:** Hege Nysted, Oda Horpestad, Ane Djuv

**Affiliations:** 1 Orthopaedic Department, Stavanger University Hospital, 4020 Stavanger, Norway; 2 Clinical Institute 1, University of Bergen, 5007 Bergen, Norway

**Keywords:** FLS, DXA, fracture, prioritising, osteoporosis, fragility fracture

## Abstract

At Stavanger University Hospital (SUH), patients aged 50 years and above with a fracture after a fall are included in our Fracture Liaison Service (FLS) at the orthopaedic department, due to their high imminent fracture risk. The FLS at SUH keeps a quality registry, including index fractures, fall from standing/walking, preventive factors, Dual Absorptiometry X-ray (DXA) results and treatment status, in addition to risk factors such as chronic diseases. As in many other hospitals and countries, the capacity of the DXA scanner at SUH does not meet the needs of the ageing population. As such, FLS patients should be prioritised for DXA scanning according to their need for anti-osteoporotic treatment. The aims of this study were (1) to identify whether any risk factors are more strongly associated with osteoporosis than others, and (2) to use this information as a tool to prioritise patients for which the decision to initiate anti-osteoporotic treatment should be assessed by a DXA scan. **Method:** We used software from CheckWare to keep a structured health record, submitting journal text to the health record and data to our fracture quality registry from 1 June 2022 to 31 December 2024. The fracture coverage of the registry, as part of the medical record, was 100%. Both men and women aged over 50 years with fragility-related fractures were included in the analysis, with index fracture having been reported within 24 months prior to FLS assessment. Exclusion criteria: short life expectancy (<3 years), already started on anti-osteoporotic treatment, living in nursing home, age >97 years, or multi-trauma patients. Statistics were calculated using SPSS and logistic regression. The results are presented as odds ratio (OR) and 95% confidence interval (95% CI). Significant differences were considered at a *p*-value of <0.05. **Results:** A total of 6974 patients were included, 81% of which were female. After the DXA scan, 5307 of the patients were started on anti-osteoporotic treatment (76%). Patients aged 50–70 years were the largest group. Female patients or those aged 80 years or older had an increased odds ratio (OR) of starting treatment after a fracture. The index fractures included in the logistic regression analysis and were most likely to initiate anti-osteoporotic treatment in the FLS, were vertebral fracture (*p* < 0.000, OR 3.1, 95% CI: 2.4–4.0), hip fracture (*p* < 0.000, OR 2.60, 95% CI: 1.9–3.5), costa fracture (*p*-value = 0.028, OR:1.3, 95% CI:1.0–1.5), pelvic fracture (*p*-value < 0.000, OR 3.1, 95% CI: 1.8–5.1). Patients with lack of sufficient vitamin D had increased odds with OR of 1.7 (*p*-value < 0.00, 95% CI: 1.3–2.2) for having osteoporosis compared to the other FLS patients. Fall from standing, walking or sitting increased the odds for osteoporosis treatment (*p*-value < 0.000, OR 2.8, 95% CI: 2.3–3.3). **Conclusions:** The listed risk factors for needing treatment were high for most fractures, especially vertebral, hip, and pelvic fractures. Patients aged 80+ years and with a fracture from standing/walking could also start treatment directly, without waiting for a DXA scan. Thus, these patients should be shifted rapidly to FLS and started on treatment without delay. In this way, DXA scanning can be prioritised for patients for whom supporting information is needed regarding the decision to initiate anti-osteoporotic treatment, such as those with proximal humerus, wrist, or ankle fractures. Time to DXA scan could be shortened for these patients and 12 weeks may be achievable.

## 1. Introduction

Osteoporosis is a progressive systemic skeletal disease characterised by low bone mass and microarchitectural deterioration of bone tissue. This can lead to increased bone fragility and susceptibility to fractures. The overarching goal of osteoporosis management is to identify fractures in patients aged 50+ years and prevent imminent fractures [1]. This approach has led to a global knowledge network known as the Fragility Fracture Network (FFN), with the International Osteoporosis Foundation (IOF) sharing a strong emphasis on the same matter; the initiatives of these organisations are captured in the phrase “Let this fracture be the last!” [2,3].

Fragility fractures are a major health concern in Europe, with millions of new cases annually. The number of cases is expected to double within the next two decades due to demographic changes, especially if preventive measures are not put into action [2]. Major osteoporotic fractures, such as those of the hips, vertebrae and pelvis, often lead to severe pain, disability and a reduction in independence, significantly affecting the elderly population [4,5,6]. The associated economic burden is substantial, with the estimated 23% increase in the number of patients—from 2.7 million patients in 2017 to 3.3 million by 2030—within the EU leading to fracture-related costs increasing from EUR 37.5 billion in 2017 to EUR 48 billion by 2030. In the same report, the disability-adjusted life years (DALYs) per 1000 individuals aged ≥50 years were estimated at 21 years, which is higher than similar estimates for stroke or chronic obstructive pulmonary disease (COPD) [7]. The costs related to both fracture management and associated healthcare services are reaching billions of euros each year and are expected to increase in the coming decades due to demographic shifts [2,8]. Therefore, finding and treating patients with osteoporotic fractures are more important than ever.

Several risk factors are associated with osteoporosis and osteoporosis-related fractures. Lifestyle factors such as smoking and excessive alcohol intake can weaken the bone structure [9]. Sedentary behaviours reduce bone strength, as do diets lacking in essential nutrients—particularly calcium and vitamin D [9,10,11]. Chronic diseases such as diabetes, hypertension and hyperthyroidism, as well as the treatments used against them can increase the risk of fracture [12,13,14]. Taking drugs such as corticosteroids (for a period longer than 3 months), antidepressants, biological immunosuppressants and antipsychotics may increase the risk of osteoporosis [12,15]. It is therefore recommended to monitor these patients with BMD measurements.

It is recommended that assessment for osteoporosis is carried out in patients aged 50 or older with a major osteoporotic fracture (MOF) as soon as possible, due to the imminent risk of subsequent fracture [3,5,16,17,18]. It is also considered to best practice to do this systematically, independent of socio-economic status, in a Fracture Liaison Service (FLS) or similar [19,20].

Fracture Liaison Services (FLSs) have been established as a crucial component of secondary fracture prevention [21]. An FLS systematically identifies, treats, and refers patients aged 50 and older who have suffered fragility fractures, aiming to reduce the risk of subsequent fractures [22]. The implementation of FLSs has led to promising results, including reduced risk of secondary fractures and lower mortality rates [3,20,23].

In an FLS it is recommended to focus on typical high-risk fractures in the hip, proximal humerus, pelvis, distal radius and vertebrae. These are referred to as major osteoporotic fractures (MOFs) [24,25,26,27]. In general, patients with MOFs should be started on treatment immediately [16]. In addition, proximal humerus fractures are commonly associated with a subsequent hip fracture within two years and should be considered for anti-osteoporotic treatment during hospital stay or within a short time (maximum 12 weeks), either at an FLS clinic or in collaboration with a primary care physician (PCP) or general practitioner (GP) [28,29]. All other fracture patients satisfying given inclusion criteria for the FLS are recommended for a Dual X-ray Absorptiometry (DXA) scan before starting treatment [30]. The availability of DXA is often scarce, with significant variability between the different European countries and others worldwide [31].

The Best Practice Framework recommends the use of an FLS registry, in order to promote quality improvement in the FLS and measure key performance indicators (KPIs) [32]. One of the KPIs in the Best Practice Framework is DXA assessment within 12 weeks after the index fracture, in order to determine whether to initiate anti-osteoporotic treatment [18,32]. Several FLSs struggle to achieve this KPI, including the Spanish FLS registry [33], and it remains the only KPI that the FLS at Stavanger University Hospital has not yet achieved [34].

## 2. Aim

As in many other hospitals and countries, the DXA scanner capacity at the SUH FLS does not meet the needs of the ageing population, especially considering the increasing lack of healthcare professionals per patient [8,35]. In view of the mismatch between the increasing number of patients in need of DXA scans and the lower number of healthcare professionals available to operate the DXA scanners, we need to prioritise the available resources in the best interests of society and the patients. In particular, those at the highest risk of starting treatment should be prioritised for immediate treatment, and not delayed in the DXA waiting queue; meanwhile, those presenting fractures identified with a lower to moderate risk should be prioritised for DXA scanning, enabling an evaluation regarding whether to initiate treatment. A patient’s risk for needing anti-osteoporotic treatment could be used to prioritise DXA scans. The aims of this study were to initially identify whether any risk factors are more strongly associated with osteoporosis than others, and then to use this information as a tool to prioritise DXA scanning for patients in doubt of needing anti-osteoporotic treatment.

## 3. Method

Data for all patients at the FLS clinic at Stavanger University Hospital are included as a structured health record (SHR), developed in a software that is available in all Norwegian hospitals (EG CheckWare, V2, Oslo, Norway). In particular, journal text was entered into the SHR and simultaneously into our Fracture registry, including the FLS quality registry [34], which has been approved by the SHR and Western health authorities in Norway (ID1404). The patients were informed about the registry, as well as the possibility of and how to withdraw their information, according to the general data protection regulation (GDPR) in the European Union. The registry, as part of the medical record system, covers 100% of fractures treated either at our outpatient emergency clinic or in the orthopaedic ward [34]. The data inclusion period was from 1 June 2022 to 31 December 2024. The medical conditions relevant to the assessment were included in the structured health record at the FLS, comprising information provided by the patients [34]. Both men and women aged over 50 years with fragility fractures are included in the registry, with index fracture reported within 24 months prior to assessment at the FLS. Associated variables include age groups (see Table 1), major osteoporotic fractures (vertebral, wrist, proximal humerus, hip, pelvic), and ankle and costa fractures. All other fractures were collected and termed “Other”, in order to maintain the anonymity of patients. Known or associated diseases or conditions which increase the risk of developing osteoporosis were included, such as autoimmune diseases (rheumatoid arthritis (RA), spondylarthritis, other systemic arthritis, ulcerative colitis (UC), Crohn’s disease, coeliac disease), endocrine diseases (diabetes mellitus I and II, hyperthyroidism), asthma or chronic obstructive pulmonary disease (COPD), gastric ulcer or gastric reflux disease (GERD), gastric bypass surgery, periodontitis or other dental disease, myocardial infarction (MI) or cardiovascular disease, ongoing cancer treatment, low vitamin D levels (<50 nmol/L), sedentary behaviour (activity less than 90 min a week), drinking alcohol > 6 units per week, cigarette use, and falling from standing or sitting. These risk factors are included in the FRAX score [25] described in the Introduction.

According to Norwegian guidelines, patients with short life expectancy (<3 years), already treated with anti-osteoporotic drugs for hip fracture at the orthopaedic ward, living in nursing home or aged >97 years can start on anti-osteoporotic treatment without a DXA scan, and were thus excluded from the analysis. The number of patients excluded due to DXA referral in the inclusion period was 78. Those patients with more than one index fracture were also excluded because of multi-trauma (178 patients). Categories including less than 10 patients were also excluded for anonymisation purposes. Data from the quality registry were handled by a responsible data manager, and were anonymised before analysis.

Statistics were calculated using SPSS Statistics (Version 26, IBM). Fisher’s Exact Test, Pearson chi-square and linear-by linear association were analysed to determine *p*-value. Significant differences were considered at a *p*-value of <0.05. Logistic regression was used, utilising the forward stepwise method with Chi-square test for covariance, including multicollinearity analysis. Covariance of less than 0.005 for the variables were accepted and the association were presented as odds ratio (OR) with 95% confidence intervals (95% CI).

## 4. Results

A total of 6974 patients were included, 81% of which were female. Of these, 5307 of the patients were started on anti-osteoporotic treatment (76%). Most of the female patients (81%, *p* < 0.001) started on treatment against osteoporosis after DXA scan. The group of patients aged 50–70 years were the largest age group, comprising 51% of the patients (see Table 1 for details). Over 90% of the patients aged 80 years or older received treatment against osteoporosis (*p*-value < 0.001). The prevalence of patients started on treatment after a vertebral fracture (*p*-value < 0.001, 91%), hip fracture (*p*-value < 0.001, 89%), pelvic fracture (*p*-value < 0.001, 91%), proximal humerus fracture (*p*-value < 0.001, 71%), wrist fracture (*p*-value = 0.002, 73%) or costa fracture (*p*-value= 0.012, 71%) was significant. Patients with costa fracture had a lower mean age than patients with vertebral, pelvic or hip fractures, with 61% aged between 50 and 70 years.

**Table 1:** Accumulated demographic data from the registry.

		Needing Treatment Against Osteoporosis?					
		No		Yes		Total Number in Each Row	*p*-Value
		Count	N%	Count	N%	N = 6974	
Gender	Female	1037	19%	4395	81%	5432	<0.001
	Male	592	39%	909	61%	1501	
Age group	50–69	1247	36%	2308	64%	3555	<0.001
	70–79	288	15%	1835	85%	2123	
	80 and older	94	8%	1164	92%	1258	

Diseases such as rheumatoid arthritis (RA), coeliac disease, diabetes mellitus I and II, asthma or chronic obstructive pulmonary disease (COPD), hyperthyroidism, ventricular ulcer or gastric reflux disease (GERD), periodontitis or other dental disease, had all significant portion of patients needing treatment against osteoporosis (*p*-value < 0.05, 72–89%) (Table 2).

Above 80% of patients with sedentary lifestyle (less than 90 min exercise weekly) started on treatment against osteoporosis, but did not have a strong association with starting anti-osteoporotic treatment compared to the other factors. Most of the patients (94%) with low body mass index (BMI) were started on treatment against osteoporosis. Cigarette smoking or drinking alcohol gave not increased odds risk for anti-osteoporotic treatment (Table 3).

**Figure 1:**
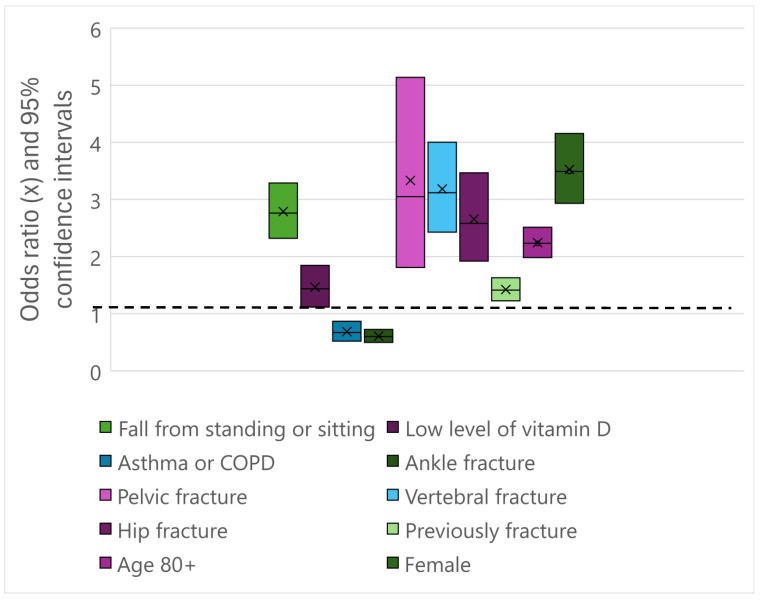
Forest diagram of the odds ratio (x) and 95% confidence intervals for the risk factors included in the registry. Fall from standing or sitting position, age 80+ years, low level of vitamin D, pelvic fracture, vertebral fracture, and hip and costa fractures were all associated with an increased risk of starting anti-osteoporotic treatment after a fracture followed by DXA. Having asthma or COPD, or ankle fractures were not associated with higher odds for starting treatment when adjusted to the other factors in our analysis.

Table 4 includes the variables included in the regression analysis. Females and patients aged 80 years or greater had an increased odds ratio (OR) for starting treatment after a fracture with OR of 3.5 (95% CI: 2.9–4.2) or 2.3 (95% CI: 2.0–2.5), respectively. Those patients who had fallen from standing, walking or sitting had increased odds for starting treatment (*p* < 0.000, OR: 2.8, 95% CI: 2.3–3.3) compared to those falling with higher energy (Table 4 and Figure 1). The index fracture types with increased OR for osteoporosis were vertebral fracture (*p* < 0.000, OR 3.1, 95% CI: 2.4–4.0), hip fracture (*p* < 0.000, OR 2.60, 95% CI: 1.9–3.5), costa fracture (*p*-value = 0.028, OR:1.3, 95% CI: 1.0–1.5) and pelvic fracture (*p* < 0.000, OR 3.1, 95% CI: 1.8–5.1). Patients with lack of sufficient vitamin D had increased odds with OR of 1.7 (*p*-value < 0.00, 95% CI: 1.3–2.2) for having osteoporosis compared to the other FLS patients (Table 4). Patients with asthma or COPD or ankle fracture had an OR of 0.7 (*p* = 0.002, 95% CI:0.52–0.87) and 0.6 (*p* < 0.000, 95% CI: 0.49–0.72), respectively (see Table 4).

## 5. Discussion

### 5.1. FLS

It is highly recommended that osteoporosis assessments are carried out in patients over the age of 50 with a fragility fracture, as stated in numerous studies [3,5,16,17,19,36]. According to the Best Practice Framework issued by the International Osteoporosis Foundation (IOF), a quality registry should be used to record quality measurements of the FLS [19,32]. In a busy hospital, such a registry may help to identify and prioritise those patients at highest risk of needing anti-osteoporotic treatment, or who need further assessment with DXA. The registration of data via a structured health record results in 100% completeness, allowing for more robust results. No patients withdrew from the registry during the inclusion period. Thus, the presented results are pragmatic and can be considered relevant to other FLSs.

Due to the high risk of needing treatment associated with hip, pelvic and vertebral fractures in patients aged over 50 years, researchers have recommended to initiating anti-osteoporotic treatment, either in the hospital or the GP setting, without any delay or need for DXA scanning [16,20,30]. The Fracture Risk Assessment Tool (FRAX tool) has been shown to correspond to the DXA-scan results and could, in these cases, be used to guide clinical decisions [25,37]. The FRAX tool is a free online tool that allows for calculation of the 10-year risk of a major osteoporotic fracture based on risk factors such as age, gender, weight, hight, smoking habits, alcohol consumption, genetic inheritance, previous fractures and bone mineral density (BMD), if available [38].

### 5.2. The Included Fractures

Major osteoporotic fractures (MOFs) are defined as fractures in the hip, proximal humerus, pelvic, distal radius or vertebrae, and the considered registry includes all types of fracture injuries. The registry identified the same high associations between osteoporosis and vertebral fracture [39], pelvic and hip fracture as in other studies [26,40]. Surprisingly, proximal humerus fractures and wrist fractures did not increase the risk for starting treatment when compared to the other fractures and risk factors included in the analysis. However, over 70% of the proximal humerus fractures and wrist fractures were started on treatment and a high prevalence of osteoporosis was observed.

About half of the ankle fractures led to the initiation of anti-osteoporotic treatment. For ankle fractures, the OR was 0.6 and, due to the relative uncertainty, a DXA scan should be performed before initiating anti-osteoporotic treatment. Most of the vertebral fractures (above 90%) initiated treatment after the DXA scan and were associated with a high OR (of 2.7–2.8). All the included index fractures had a significant anti-osteoporotic treatment prevalence of in the range of 55–91% (see Table 2). Except from the ankle fractures, all the fractures included were associated with significantly increased odds ratio (ranging from 1.8 to 2.2) for initiating anti-osteoporotic treatment (Table 4 and Figure 1). The importance of included all fracture patients in the FLS is confirmed with these numbers and are in line with other papers [2,25,41,42].

The most frail patients—those living in nursing homes—started anti-osteoporotic treatment at the hospital or the nursing home due to their high risk, according to the guidelines issued by the Norwegian orthopaedic association [30]. Prioritising these high-risk fractures for immediate treatment decreases the DXA waiting time for the remaining patients. One can argue that those with lower association for osteoporosis but who are still at high risk should be prioritised for DXA scanning; e.g., those with proximal humerus, wrist and ankle fractures. A recent study recommended starting treatment in-hospital with proximal humerus fracture as the index fracture, due to the imminent risk of hip fracture [18]. For patients with aged 80 years or older, this could be argued from our registry numbers.

Costa fractures are not commonly considered to be a major osteoporotic fracture (MOF) [25,41]. In the registry, we found that 73% of patients with these fractures needed treatment; they had a lower mean age than those with vertebral, pelvic and hip fractures, with 61% aged between 50 and 70 years; and 30% were male. This is in line with results from an artificial intelligence (AI)-based study of chest CTs from a trauma centre including all adults with a costa fracture [43]. Therefore, costa fracture patients should be identified and prioritised for DXA scanning due to their younger age and high risk of osteoporosis as a cause of fracture; thereby indicating a higher subsequent fracture risk.

### 5.3. Medical Conditions as Risk Factors

Vitamin D level below 50 nmol/L was associated with an increased risk of initiating anti-osteoporotic treatment after a fracture followed by DXA [41]. The optimal level of vitamin D has been debated for a long time, and different treatment thresholds have been suggested [44,45]. Our numbers indicated that levels below <50 nmol/L should be avoided for fracture patients or patients at risk for MOFs.

Increased risk in patients with periodontitis or rheumatoid arthritis has previously been reported to be associated with osteoporosis [46,47]. Systematic inflammation has been identified as a possible cause of reduced bone mineralisation, and treatment via injection of zoledronic acid improved the associated symptoms [46,48,49]. Although our numbers did not reveal a significant increase in risk compared to the other risk factors or the index fracture, over 80% of those patients having a diagnosis of RA or periodontitis, were started on anti-osteoporotic treatment after a fracture.

Having asthma or chronic obstructive pulmonary disease (COPD) did not increased the risk for osteoporosis in our FLS population. A meta-analysis have shown an increased risk of osteoporosis in COPD patients compared to healthy controls, but the study populations included are not similar to the FLS patients [50]. Nearly 90% of patients with diabetes mellitus type I or II started with anti-osteoporotic treatment after DXA. However, diabetes mellitus type I or II was not associated with higher odds for starting treatment, when adjusted to the other covariates in the FLS population. This finding corresponds to findings by Starup-Linde et al. [51] and a DXA scan would be recommended.

### 5.4. Lifestyle and Ageing Risk Factors

Having a fall from low height, such as from standing, walking or sitting, added a significant increased risk of initiating anti-osteoporotic treatment. The risk was 2.8 higher, compared to falls from heights or high-energy trauma. However, most of the patients were in this group (84%) and, so, the variation in this group must be taken into consideration with regard to priority in the DXA queue. It has been previously reported that trauma energy appears to be of little importance [5]. In addition, the combined effects of diseases and drugs might add to the risk of both falling from a standing or sitting position and osteoporosis [52].

Sedentary lifestyle, indicated by less than 90 min exercise weekly, did not have a strong association with starting anti-osteoporotic treatment. Self-reported high alcohol consumption also gave no extra risk of needing treatment, compared to the other risk factors from the analysis; rather, it seemed to protect the patients. One can argue that the patients with alcohol abuse in their history would not report this in the same manner as those with higher socioeconomic status associated with a more modest drinking pattern and, therefore, might have other protective factors against osteoporosis, as reported in other studies [53,54]. This illustrates the limitations of using registry data on sensitive questions such as personal alcohol use, and the results should be interpreted with caution.

Fracture in combination with fall from standing or age above 80 years were both strongly associated with initiation of anti-osteoporotic treatment. One might argue that all patients with age above 80 years and/or fracture after fall from a low height, such from standing, walking or sitting, could be started on treatment immediately without waiting for DXA—especially those with the MOFs listed above. This is in line with other recommendations [4,6,25,39,55].

### 5.5. Clinical Relevance

The most efficient FLS model—and the one recommended in the Best Practice Framework issued by the IOF—includes the key elements of identification, investigation, intervention and adherence to follow-up. In the highly recommended FLS pathway, patients receive treatment without any delay after the DXA scan, and the treatment is followed up [16,32,42]. At SUH, the FLS is organised by an orthopaedic surgeon who is responsible for the prescription of medical treatments; they work about 6–8 h per week and review about 90 patient charts after DXA scans performed by FLS nurses. A second model includes the GP or primary care (PC), who can initiate treatment after the DXA scan performed at the FLS. The third model involves providing the patient and the GP/PC information regarding the risk of osteoporosis after a fracture for those aged 50+, as well as conditions for the GP/PC to refer patients for a DXA scan. One might increase the care gap through the two latter models, as it depends much more on the patient capacity, health compliance, and interest and competence of the GP/PC with respect to secondary osteoporosis and fracture prevention [16]. To the best of our knowledge, a study with a similar number of patients, covering all of those with a fracture in a large orthopaedic trauma hospital with 100% completeness, has not been published previously.

In the inclusion period (1 June 2022 to 31 December 2024), the FLS centre at SUH possessed at least one DXA scanner; however, in a one-year sub-period (1 March 2022 to 1 March 2023), two operational DXA scanners and two to three FLS nurses were available. The DXA scanner capacity per capita ranged from 2.6 to 5.3 per million inhabitants at SUH, which is far below the average in EU countries [31,56]: in the paper “Bone densitometry worldwide: a global survey by the ISCD and IOF,” a global map of the DXA scanners and FLS distribution has been provided, indicating the wide variation in DXA scanner capacity per capita [56].

The Spanish FLS registry, Spanish National Registry of Major Osteoporotic Fractures (REFRA), included 2965 patients in 2022 [33]. REFRA include MOFs in their registry and have also reported a lack of DXA scanner capacity. The Australian and New Zealand Fragility Fracture Registry (ANZFFR) reported a DXA scan rate within 16 weeks of 25% in 2023–2024 and a capture rate of 72% [57]. Thus, more than 75% of patients did not receive a DXA scan within 16 weeks in New Zealand and Australia in 2023–2024.

As several other countries or hospitals around the world are experiencing the same mismatch between the increasing amount of elderly individuals with a fracture and the lack of DXA facilities and healthcare professionals [7], our study focused on which patients to prioritise based on the fracture type and other risk factors. Such prioritisation is crucial, as no more resources are in sight within the coming decades.

## 6. Limitations

The study is a prospective registry study using data from patients having a fracture treated and aged 50 years or older in the southwest part of Norway, at Stavanger University Hospital. A single-centre hospital registry is only available at present. The hospital is the sole hospital treating fractures in an area with a population of about 400,000 inhabitants and is the third-largest orthopaedic trauma hospital in Norway. Although all the included subgroups had above 10 patients, the numbers were too low in some groups (e.g., those with ulcerative colitis) to obtain meaningful conclusions. The limited statistical power for small subgroups must be considered when drawing conclusions.

Assessment, treatment and follow-up are cheap for patients in Norway, and are free if they have exceeded about EUR 300 in health expenses per year. Thus, the obtained results might not be relevant to other countries in the world.

## 7. Conclusions

A patient’s risk for needing anti-osteoporotic treatment could be used to prioritise DXA scans. The aims of this study were to initially identify whether any risk factors are more strongly associated with osteoporosis than others, and then to use this information as a tool to prioritise DXA scanning for patients in doubt of needing anti-osteoporotic treatment.

The listed risk factors for needing treatment against osteoporosis were high and especially for vertebral, hip and pelvic fractures. In addition, patients aged 80+ years, low-D vitamin level or with a fracture from standing/walking had increased risk and could start treatment directly, without waiting for a DXA scan. Thus, these patients should be shifted rapidly to FLS and started on treatment without delay. In this way, DXA scanning can be prioritised for patients for whom supporting information is needed regarding the decision to initiate anti-osteoporotic treatment, such as those with proximal humerus, wrist or ankle fractures. As a result, time to DXA scan could be shortened for these patients and 12 weeks may be achievable.

## Figures and Tables

**Table 2:** Conditions serving as risk factors for fracture in addition to a fracture. Abbreviations: chronic obstructive pulmonary disease (COPD), ulcerative colitis (UC), and gastric reflux disease (GERD).

Diseases and Fractures:	Need Treatment Against Osteoporosis				
	No		Yes		
	Count	N%	Count	N%	*p*-Value
Rheumatoid arthritis	52	15%	295	85%	<0.001
Other systemic arthritis	55	14%	150	86%	0.008
Coeliac disease	18	14%	105	86%	0.003
Spondylarthritis	25	27%	67	73%	0.234
Ulcerative colitis	12	23%	41	77%	0.518
Crohn’s disease	22	27%	57	73%	0.213
Diabetes mellitus I and II	37	11%	292	89%	<0.001
Asthma or COPD	171	28%	447	72%	0.006
Hyperthyroidism	194	19%	853	81%	<0.001
Ventricular ulcer or GERD	20	14%	118	86%	0.006
Periodontitis or other dental disease	132	16%	692	84%	<0.001
Vertebral fracture	93	9%	917	91%	<0.001
Wrist fracture	381	27%	1009	73%	0.002
Proximal humerus fracture	185	29%	456	71%	<0.001
Ankle fracture	387	45%	478	55%	<0.001
Hip fracture	71	11%	579	89%	<0.001
Pelvic fracture	26	9%	268	91%	<0.001
Costa fracture	242	27%	670	73%	0.012

**Table 3:** Lifestyle factors associated with increased risk of osteoporosis included in the register.

Lifestyle Risks:		Need Anti-Osteoporotic Treatment?				
		No		Yes		
		Count	N%	Count	N%	*p*-Value
Fall from standing or sitting?	No	414	47%	470	53%	<0.001
	Yes	969	20%	3774	80%	
Performing activity for 90 min or more (exercise/walk) a week?	No	1506	24%	4709	76%	<0.001
	Yes	123	17%	598	83%	
Exercising strength or balance for a minimum of 90 min per week?	No	1362	23%	4436	77%	0.509
	Yes	267	23%	871	77%	
Drinking alcohol	No	1023	21%	3874	79%	<0.001
	Yes	606	30%	1433	70%	
Drinking alcohol ≥6 units per week?	No	1309	24%	4127	76%	<0.001
	Yes	74	39%	117	61%	
Cigarette smoking	No	711	24%	2302	76%	0.435
	Yes	918	23%	3005	77%	
Adequate vitamin D values (<50 nmol/L)?	No	111	37%	189	63%	<0.001
	Yes	955	23%	3243	77%	
Previous fracture before index fracture?	No	745	29%	1828	71%	<0.001
	Yes	611	20%	2443	80%	
Body mass index (BMI)	Normal	954	19%	3985	81%	<0.001
	Low	8	6%	119	94%	
	Obese	526	42%	728	58%	

**Table 4:** Risk for starting treatment after DXA scan in the FLS, corrected for type of fracture, diseases and other risk factors in the logistic regression analysis. Abbreviations: chronic obstructive pulmonary disease (COPD), odds ratio (OR), confidence interval (CI).

Included Variables	OR	95% C.I. for OR		*p*-Value
		Lower	Upper	
Age 80+	2.23	1.981	2.513	0.000
Female gender	3.49	2.932	4.155	0.000
Vertebral fracture	3.12	2.428	4.002	0.000
Ankle fracture	0.60	0.495	0.724	0.000
Hip fracture	2.60	1.920	3.467	0.000
Pelvic fracture	3.05	1.809	5.138	0.000
Costa fracture	1.26	1.025	1.544	0.028
Previously fracture	1.41	1.225	1.629	0.000
Asthma or COPD	0.67	0.518	0.867	0.002
Low level of vitamin D	1.43	1.115	1.846	0.005
Fall from standing, walking or sitting	2.76	2.319	3.288	0.000

## Data Availability

The original contributions presented in the study are included in the article, further inquiries can be directed to the corresponding author.

## References

[1] Cosman F., Lewiecki E.M., Eastell R., Ebeling P.R., De Beur S.J., Langdahl B., Rhee Y., Fuleihan G.E., Kiel D.P., Schousboe J.T. (2024). Goal-directed osteoporosis treatment: ASBMR/BHOF task force position statement 2024. J. Bone Min. Res..

[2] Kanis J.A., Norton N., Harvey N.C., Jacobson T., Johansson H., Lorentzon M., McCloskey E.V., Willers C., Borgstrom F. (2021). SCOPE 2021: A new scorecard for osteoporosis in Europe. Arch. Osteoporos..

[3] Akesson K., Marsh D., Mitchell P.J., McLellan A.R., Stenmark J., Pierroz D.D., Kyer C., Cooper C. (2013). Capture the Fracture: A Best Practice Framework and global campaign to break the fragility fracture cycle. Osteoporos. Int..

[4] Borgstrom F., Lorentzon M., Johansson H., Harvey N.C., McCloskey E., Willems D., Knutsson D. (2024). Cost-effectiveness intervention thresholds for romosozumab and teriparatide in the treatment of osteoporosis in the UK. Osteoporos. Int..

[5] Leslie W.D., Schousboe J.T., Morin S.N., Martineau P., Lix L.M., Johansson H., McCloskey E.V., Harvey N.C., Kanis J.A. (2020). Fracture risk following high-trauma versus low-trauma fracture: A registry-based cohort study. Osteoporos. Int..

[6] Skjodt M.K., Nicolaes J., Smith C.D., Olsen K.R., Cooper C., Libanati C., Abrahamsen B. (2023). Fracture Risk in Men and Women with Vertebral Fractures Identified Opportunistically on Routine Computed Tomography Scans and Not Treated for Osteoporosis: An Observational Cohort Study. J. Bone Miner. Res. Plus.

[7] Borgstrom F., Karlsson L., Ortsater G., Norton N., Halbout P., Cooper C., Lorentzon M., McCloskey E.V., Harvey N.C., Javaid M.K. (2020). Fragility fractures in Europe: Burden, management and opportunities. Arch. Osteoporos..

[8] Hernlund E., Svedbom A., Ivergard M., Compston J., Cooper C., Stenmark J., McCloskey E.V., Jonsson B., Kanis J.A. (2013). Osteoporosis in the European Union: Medical management, epidemiology and economic burden. A report prepared in collaboration with the International Osteoporosis Foundation (IOF) and the European Federation of Pharmaceutical Industry Associations (EFPIA). Arch. Osteoporos..

[9] Weaver C.M., Gordon C.M., Janz K.F., Kalkwarf H.J., Lappe J.M., Lewis R., O’Karma M., Wallace T.C., Zemel B.S. (2016). The National Osteoporosis Foundation’s position statement on peak bone mass development and lifestyle factors: A systematic review and implementation recommendations. Osteoporos. Int..

[10] Ishimi Y. (2015). Osteoporosis and Lifestyle. J. Nutr. Sci. Vitaminol..

[11] Abdolalipour S., Mirghafourvand M., Ghassab-Abdollahi N., Farshbaf-Khalili A. (2021). Health-promoting lifestyle and quality of life in affected and unaffected menopausal women by primary osteoporosis. J. Educ. Health Promot..

[12] Vestergaard P., Rejnmark L., Mosekilde L. (2006). Methotrexate, azathioprine, cyclosporine, and risk of fracture. Calcif. Tissue Int..

[13] Subarajan P., Arceo-Mendoza R.M., Camacho P.M. (2024). Postmenopausal Osteoporosis: A Review of Latest Guidelines. Endocrinol. Metab. Clin. N. Am..

[14] Lems W.F. (2017). Is Fragility Fracture a Strong Risk Factor for a Cardiovascular Event in Rheumatoid Arthritis? The Challenge of Dealing with Multiple Comorbidities. J. Rheumatol..

[15] Almeida O.P., Page A., Sanfilippo F.M., Etherton-Beer C. (2024). Prospective Association Between the Dispensing of Antidepressants and of Medications to Treat Osteoporosis in Older Age. Am. J. Geriatr. Psychiatry.

[16] Akesson K.E., McGuigan F.E.A. (2021). Closing the Osteoporosis Care Gap. Curr. Osteoporos. Rep..

[17] Mackey D.C., Lui L.Y., Cawthon P.M., Bauer D.C., Nevitt M.C., Cauley J.A., Hillier T.A., Lewis C.E., Barrett-Connor E., Cummings S.R. (2007). High-trauma fractures and low bone mineral density in older women and men. JAMA.

[18] Toth E., Banefelt J., Akesson K., Spangeus A., Ortsater G., Libanati C. (2020). History of Previous Fracture and Imminent Fracture Risk in Swedish Women Aged 55 to 90 Years Presenting with a Fragility Fracture. J. Bone Miner. Res..

[19] Javaid M.K., Kyer C., Mitchell P.J., Chana J., Moss C., Edwards M.H., McLellan A.R., Stenmark J., Pierroz D.D., Schneider M.C. (2015). Effective secondary fracture prevention: Implementation of a global benchmarking of clinical quality using the IOF Capture the Fracture(R) Best Practice Framework tool. Osteoporos. Int..

[20] Andreasen C., Dahl C., Frihagen F., Borgen T.T., Basso T., Gjertsen J.E., Figved W., Wisloff T., Hagen G., Apalset E.M. (2025). Fracture liaison service (FLS) is associated with lower subsequent fragility fracture risk and mortality: NoFRACT (the Norwegian capture the fracture initiative). Osteoporos. Int..

[21] McLellan A.R., Wolowacz S.E., Zimovetz E.A., Beard S.M., Lock S., McCrink L., Adekunle F., Roberts D. (2011). Fracture liaison services for the evaluation and management of patients with osteoporotic fracture: A cost-effectiveness evaluation based on data collected over 8 years of service provision. Osteoporos. Int..

[22] Chesser T.J.S., Javaid M.K., Mohsin Z., Pari C., Belluati A., Contini A., Caiaffa V., Chana-Rodriguez F., Gomez-Vallejo J., Sanchez-Perez C. (2022). Overview of fracture liaison services in the UK and Europe: Standards, model of care, funding, and challenges. OTA Int..

[23] Dell R. (2011). Fracture prevention in Kaiser Permanente Southern California. Osteoporos. Int..

[24] Lv H., Nie Y., Wang X., Li W., Wang Y., Li Z., Zhang X., Chen W. (2022). Epidemiological characteristics of fractures of spine, hip, proximal humerus and forearm during the haze epidemic period. Injury.

[25] Kanis J.A., Johansson H., McCloskey E.V., Liu E., Akesson K.E., Anderson F.A., Azagra R., Bager C.L., Beaudart C., Bischoff-Ferrari H.A. (2023). Previous fracture and subsequent fracture risk: A meta-analysis to update FRAX. Osteoporos. Int..

[26] Johansen A., Sahota O., Dockery F., Black A.J., MacLullich A.M.J., Javaid M.K., Ahern E., Gregson C.L. (2023). Call to action: A five nations consensus on the use of intravenous zoledronate after hip fracture. Age Ageing.

[27] NICE (2017). Osteoporosis: Assessing the Risk of Fragility Fracture. Clinical Guideline.

[28] Haque A., Singh H.P. (2020). Mortality following combined fractures of the hip and proximal humerus. World J. Orthop..

[29] Curtin P.B., Hall R.R., Molla V.G., Lansbury J.N., O’Connor E.P., Aaron D.L. (2022). Morbidity and mortality of fragility proximal humerus fractures: A retrospective cohort study of patients presenting to a level one trauma center. J. Shoulder Elb. Surg..

[30] Frihagen F., Djuv A., Nordbø J.V., Solberg L.B. (2022). Treatment Guide for Fragility Fractures in Norway (Behandlingsveileder ved Lavenergibrudd). https://www.legeforeningen.no/foreningsledd/fagmed/norsk-ortopedisk-forening/faggrupper/faggruppe-for-osteoporose-og-benhelse/behandlingsveileder-ved-lavenergibrudd/.

[31] Kanis J.A., Johnell O. (2005). Requirements for DXA for the management of osteoporosis in Europe. Osteoporos. Int..

[32] Javaid M.K., Sami A., Lems W., Mitchell P., Thomas T., Singer A., Speerin R., Fujita M., Pierroz D.D., Akesson K. (2020). A patient-level key performance indicator set to measure the effectiveness of fracture liaison services and guide quality improvement: A position paper of the IOF Capture the Fracture Working Group, National Osteoporosis Foundation and Fragility Fracture Network. Osteoporos. Int..

[33] Montoya-Garcia M.J., Carbonell-Abella C., Cancio-Trujillo J.M., Moro-Alvarez M.J., Mora-Fernandez J., Izquierdo-Avino R., Nogues X., Mesa-Ramos M., Segundo-Mozo R.M.S., Calero-Munoz E. (2022). Spanish National Registry of Major Osteoporotic Fractures (REFRA) seen at Fracture Liaison Services (FLS): Objectives and quality standards. Arch. Osteoporos..

[34] Djuv A., Harboe K., Nysted H., Kirkhus T.K., Horpestad O., Birkeland F.H., Mehl B.W., Johnsen E., Paulsen A. (2023). Improving the quality of the fracture liaison service through the implementation of a structured health record. BMJ Open Qual..

[35] Jones C.H., Dolsten M. (2024). Author Correction: Healthcare on the brink: Navigating the challenges of an aging society in the United States. npj Aging.

[36] Leal J., Gray A.M., Hawley S., Prieto-Alhambra D., Delmestri A., Arden N.K., Cooper C., Javaid M.K., Judge A., The REFReSH Study Group (2017). Cost-Effectiveness of Orthogeriatric and Fracture Liaison Service Models of Care for Hip Fracture Patients: A Population-Based Study. J. Bone Miner. Res..

[37] Leslie W.D., Majumdar S.R., Lix L.M., Johansson H., Oden A., McCloskey E., Kanis J.A. (2012). High fracture probability with FRAX usually indicates densitometric osteoporosis: Implications for clinical practice. Osteoporos. Int..

[38] Kanis J.A., McCloskey E., Johansson H., Oden A., Leslie W.D. (2012). FRAX((R)) with and without bone mineral density. Calcif. Tissue Int..

[39] Lems W.F., Paccou J., Zhang J., Fuggle N.R., Chandran M., Harvey N.C., Cooper C., Javaid K., Ferrari S., Akesson K.E.G. (2021). International Osteoporosis Foundation Fracture Working, Vertebral fracture: Epidemiology, impact and use of DXA vertebral fracture assessment in fracture liaison services. Osteoporos. Int..

[40] Kannus P., Palvanen M., Niemi S., Parkkari J., Jarvinen M. (2000). Epidemiology of osteoporotic pelvic fractures in elderly people in Finland: Sharp increase in 1970–1997 and alarming projections for the new millennium. Osteoporos. Int..

[41] Pisani P., Renna M.D., Conversano F., Casciaro E., Di Paola M., Quarta E., Muratore M., Casciaro S. (2016). Major osteoporotic fragility fractures: Risk factor updates and societal impact. World J. Orthop..

[42] Chandran M., Akesson K.E., Javaid M.K., Harvey N., Blank R.D., Brandi M.L., Chevalley T., Cinelli P., Cooper C., Lems W. (2024). Impact of osteoporosis and osteoporosis medications on fracture healing: A narrative review. Osteoporos. Int..

[43] Tang Y., Hong W., Xu X., Li M., Jin L. (2023). Traumatic rib fracture patterns associated with bone mineral density statuses derived from CT images. Front. Endocrinol..

[44] Reid I.R., Bolland M.J. (2020). Calcium and/or Vitamin D Supplementation for the Prevention of Fragility Fractures: Who Needs It?. Nutrients.

[45] Bendik I., Friedel A., Roos F.F., Weber P., Eggersdorfer M. (2014). Vitamin D: A critical and essential micronutrient for human health. Front. Physiol..

[46] Wang C.J., McCauley L.K. (2016). Osteoporosis and Periodontitis. Curr. Osteoporos. Rep..

[47] Gupta N., Kanwar N., Arora A., Khatri K., Kanwal A. (2024). The interplay of rheumatoid arthritis and osteoporosis: Exploring the pathogenesis and pharmacological approaches. Clin. Rheumatol..

[48] Xu G., Guo Y., Wang W., Yu W.Q., Chen Q.M., Wang H. (2024). Zoledronic acid improves periodontal health, reduces serum inflammation, and enhances bone metabolism in postmenopausal osteoporosis complicated by periodontitis. Am. J. Transl. Res..

[49] Miyamoto T. (2025). Osteoporosis and Rheumatoid Arthritis: Mechanisms Underlying Osteoclast Differentiation and Activation or Factors Associated with Hip Fractures. J. Clin. Med..

[50] Chen Y.W., Ramsook A.H., Coxson H.O., Bon J., Reid W.D. (2019). Prevalence and Risk Factors for Osteoporosis in Individuals with COPD: A Systematic Review and Meta-analysis. Chest.

[51] Starup-Linde J., Stoy J., Grinderslev P.B., Langdahl B., Harslof T. (2025). Prevalence and risk factors for osteoporosis in type 1 diabetes-results from an observational study. Osteoporos. Int..

[52] Alarkawi D., Tran T.S., Chen W., March L.M., Blyth F.M., Blank R.D., Bliuc D., Center J.R. (2024). Health Perceptions, Multimorbidity, and New Fractures and Mortality Among Patients with a Fracture. JAMA Netw. Open.

[53] Gebrelibanos M.B. (2023). The Association Between Alcohol Consumption and Self-Rated Health Among Adult Norwegian Women 2023. Master’s Thesis.

[54] Devaux M., Sassi F. (2016). Social disparities in hazardous alcohol use: Self-report bias may lead to incorrect estimates. Eur. J. Public Health.

[55] Eastell R., Rosen C.J., Black D.M., Cheung A.M., Murad M.H., Shoback D. (2019). Pharmacological Management of Osteoporosis in Postmenopausal Women: An Endocrine Society* Clinical Practice Guideline. J. Clin. Endocrinol. Metab..

[56] Clynes M.A., Westbury L.D., Dennison E.M., Kanis J.A., Javaid M.K., Harvey N.C., Fujita M., Cooper C., Leslie W.D., Shuhart C.R. (2020). Bone densitometry worldwide: A global survey by the ISCD and IOF. Osteoporos. Int..

[57] Australian and New Zealand Fragility Fracture Registry (2025). 2025 ANZFFR Annual Report. https://fragilityfracture.co.nz/2025-annual-report/.

